# Bias and inference from misspecified mixed‐effect models in stepped wedge trial analysis

**DOI:** 10.1002/sim.7348

**Published:** 2017-05-28

**Authors:** Jennifer A. Thompson, Katherine L. Fielding, Calum Davey, Alexander M. Aiken, James R. Hargreaves, Richard J. Hayes

**Affiliations:** ^1^ Department of Infectious Disease Epidemiology London School of Hygiene and Tropical Medicine London U.K.; ^2^ MRC London Hub for Trials Methodology Research London U.K.; ^3^ Department of Social and Environmental Health Research London School of Hygiene and Tropical Medicine London U.K.

**Keywords:** stepped wedge trials, cluster randomised trials, mixed‐effect model, model misspecification, simulation study

## Abstract

Many stepped wedge trials (SWTs) are analysed by using a mixed‐effect model with a random intercept and fixed effects for the intervention and time periods (referred to here as the standard model). However, it is not known whether this model is robust to misspecification.

We simulated SWTs with three groups of clusters and two time periods; one group received the intervention during the first period and two groups in the second period. We simulated period and intervention effects that were either common‐to‐all or varied‐between clusters. Data were analysed with the standard model or with additional random effects for period effect or intervention effect. In a second simulation study, we explored the weight given to within‐cluster comparisons by simulating a larger intervention effect in the group of the trial that experienced both the control and intervention conditions and applying the three analysis models described previously.

Across 500 simulations, we computed bias and confidence interval coverage of the estimated intervention effect.

We found up to 50% bias in intervention effect estimates when period or intervention effects varied between clusters and were treated as fixed effects in the analysis. All misspecified models showed undercoverage of 95% confidence intervals, particularly the standard model. A large weight was given to within‐cluster comparisons in the standard model.

In the SWTs simulated here, mixed‐effect models were highly sensitive to departures from the model assumptions, which can be explained by the high dependence on within‐cluster comparisons. Trialists should consider including a random effect for time period in their SWT analysis model. © 2017 The Authors. *Statistics in Medicine* published by John Wiley & Sons Ltd.

## 1 Introduction

Recent reanalysis of a high‐profile stepped wedge trial (SWT) has brought into question methods commonly used to analyse these complex studies 1, 2, 3. SWTs are often analysed by using models that make strong assumptions about the clustering in the data 4. It is currently unknown if estimates from these models are robust to deviations from these assumptions.

An SWT is a type of cluster randomised trial where clusters are randomised into groups. Each group begins to receive the intervention at a different time so that all clusters start the trial in the control condition, and by the end of the trial, all clusters are receiving the intervention.

The control and intervention conditions can, in principle, be compared in two directions known as the vertical and horizontal comparisons 4. Vertical comparisons compare the outcomes of clusters in the intervention condition with the outcomes of clusters in the control condition within the same time period; because the order of rollout is randomised, each of these comparisons is randomised. Horizontal comparisons compare outcomes from periods in the intervention condition with outcomes from periods in the control condition in the same cluster; these are non‐randomised before–after comparisons that are confounded with time period.

In practice, most analysis methods for SWTs incorporate information from both the vertical and horizontal comparisons in the intervention effect estimate and so need some way to adjust for period effects 4. The most common analysis model (hereafter referred to as the standard model) is a mixed‐effect model with a random intercept to account for clustering and adjusting for period effects as a fixed categorical variable; this model is described by Hussey and Hughes 5. Despite its wide use, guidance for using this analysis model is lacking. The model makes strong assumptions about the correlation structure of the data: The intervention effect and the period effects are assumed to be common to all clusters. It is not currently known whether the intervention effect estimate and its precision are robust to misspecifying these assumptions.

In the context of SWTs, we are most interested in estimation of the intervention effect and how robust this effect is to misspecification of the intervention effect itself as well as misspecification of the period effect. Previous research has found that misspecifying the random effects led to biased effect estimates as well as biased precision of estimates 6. In parallel cluster randomised trials with baseline measurements and in cluster crossover randomised trials, it has been shown that analyses with hierarchical models should include a random effect for period, sometimes referred to as a cluster‐period interaction, to avoid residual confounding 7, 8, 9, 10.

The importance of specifying the period effect correctly will depend on how much the horizontal comparisons contribute within the model. This has not been explored in the literature. If a large weight is given to this comparison, any residual confounding of the intervention effect by the period effects could lead to a biased estimate of the intervention effect.

In this paper, we will explore both issues with a simulation study comparing the standard model with other mixed‐effect models, focusing on a binary outcome with cross‐sectional measurements. We then run a second set of simulations to explore the weight given to horizontal comparisons by each analysis model. Following the simulation studies, we explore the impact of misspecifying analysis models in our motivating example.

## 2 Motivating example

**Figure 1:**
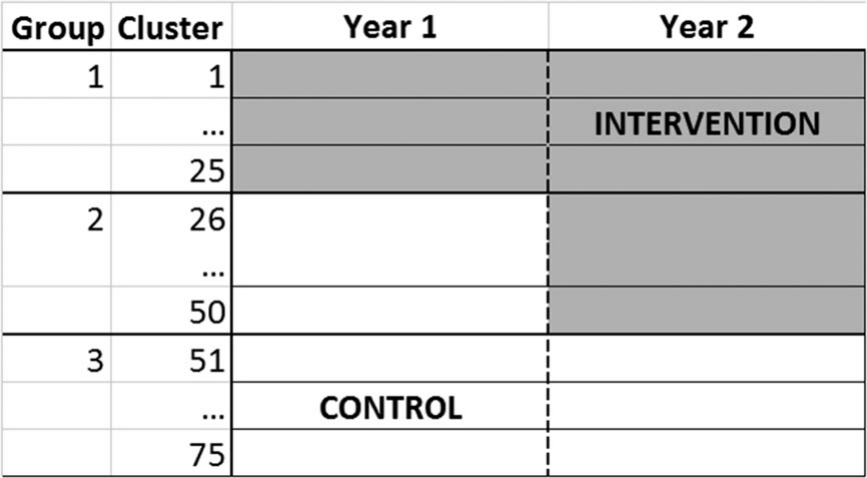
Schematic of motivating example: A stepped wedge trial (SWT) with 75 clusters randomised to three groups. The trial consisted of two time periods (years). Group 1 switched to the intervention at the start of period 1. Group 2 switched to the intervention at the start of period 2. Group 3 did not switch to the intervention.

There has been much debate in recent literature about the results of a reanalysis of a highly cited SWT that investigated the effect on school attendance of a mass deworming intervention for school children in Kenya 1, 2, 3. The trial included 75 schools (clusters) that were randomised into three groups and ran over 2 years. School attendance was measured as a binary outcome with multiple observations for each individual child during each year. There was a geometric mean of 1180 (interquartile range (IQR) 908.5, 1864) observations in each school each year, with the attendance assessed on the same children in year 2 as year 1. Children from schools in the first group began receiving the intervention at the start of the first year. Children from schools in the second group received no intervention during the first year and began receiving the intervention in the second year of the study. Children from schools in the third group did not receive the intervention during these two years (Figure 1).

In the reanalysis of this trial, it was found that the odds ratios (ORs) for school attendance for year 1 and year 2 were both smaller when analysed individually (OR = 1.48 and 1.23 respectively) than the OR given by the standard model when the data were pooled from both years (OR = 1.82) 2. We hypothesised that this could have been because the analysis model was misspecified and explored two potential types of misspecification:

1. The period effects varied between clusters. The standard model assumes that the period effects are common to all clusters. This could lead to a biased estimation of the intervention effect through biased estimation of the period effects.
2. The intervention effect varied between clusters. The standard model assumes that the intervention effect is common to all clusters. Treating an effect that truly varies as a fixed effect has been shown to lead to biased estimation of that covariate 6, and so the estimate of the intervention effect could be biased.

In this paper, we first used a simulation study based on the motivating example to explore the effect of ignoring variability between the clusters in the period effect and intervention effect in the analysis of SWTs. Second, we hypothesised that the effect of misspecification would by highly influenced by the weight given to horizontal comparisons in each analysis model and so also performed a further set of simulations to investigate this question. We then analysed the motivating example with different analysis models and compared the results in light of the findings of the simulation studies.

## 3 Simulation study methods

### 3.1 Simulation study 1

To investigate the impact of ignoring heterogeneity between clusters in the period effect and intervention effect, we compared analysis models that assumed these effects were common to all clusters (the standard model) to analysis models which allowed these effects to vary between clusters. We performed this with data in which the true underlying period effect and intervention effect were either common to all clusters or varied between clusters. A description of the scenarios we used to compare the analysis models is given, followed by the three analysis models we compared. A summary of the data scenarios simulated is given in Table 1.

**Table 1:** Summary of simulation study data scenarios.

	Description	Similar to motivating example?
Common to all simulations		
Number of groups	3	Yes
Number of time periods	2. In period 1, group 1 received the intervention. In period 2, groups 1 and 2 received the intervention.	Yes
Number of clusters	75	Yes
Cluster size	Log‐normal(6.9, 0.74) in each year. Geometric mean = 1027	Yes
Correlation of measurements within clusters	Independent within cluster‐periods	No
Mean outcome in year 1	Odds = 6.61	Yes
Mean change in outcome from year 1 to year 2	Odds ratio = 0.32	Yes
Different scenarios		
Period effect	(1) Common period effect, high variability	No
	(2) Common period effect, low variability	No
	(3) Varying period effect, decreasing variability	Yes
	(4) Varying period effect, stable variability	No
Intervention effect	(a) Log(OR) = 0.41 common to all clusters	No
	(b) Log(OR) = 0.41, varying between clusters	No
Intervention effect in group 2		
Simulation study 1	Intervention effect in group 2 the same as group 1 log(OR) = 0.41	No
Simulation study 2	Intervention effect in group 2 is log(OR) = 1.5, and group 1 is log(OR) = 0.41	No

We used the same trial design as our motivating example with clusters randomised into three groups and followed for two time periods. During the first period, only the first group had received the intervention, and during the second time period, the first and second groups had received the intervention. The third group never received the intervention. This trial design was chosen due to its simplicity; because there are only two time periods, the period effect is simple to model. The horizontal comparison is only possible in one group; this allowed us to explore the weight given to this comparison. To mimic the motivating example and to avoid issues with small sample size, we assigned 25 clusters to each group and the number of observations in a cluster in each time period was drawn from a log‐normal distribution (*μ* = 6.9, *σ* = 0.74); this gave a geometric mean number of observations in each cluster in each time period of 1027 (IQR 669, 1798).

**Figure 2:**
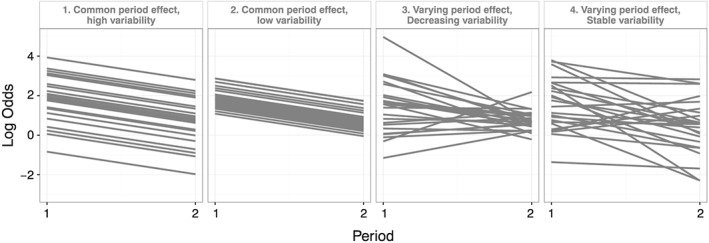
Simulated cluster‐level log odds in each period effect scenario. A sample of 25 clusters is shown in time periods 1 and 2. All are in control condition.

The cluster‐level distribution of the outcome in the first period and the change from period 1 to period 2 (the period effect) was based on group 3 of the motivating example. This group was chosen because it did not receive the intervention. We modelled the log‐odds in the first period and the log‐OR period effect from the motivating example as a bivariate normal distribution. This gave mean values for the log‐odds in period 1 and log‐OR period effect, together with a 2 × 2 covariance matrix. This distribution described the outcome and how it varied between the clusters in each period. The mean values were used in all the simulation scenarios, but we manipulated the covariance matrix to create four scenarios of how the outcome varied between the clusters and periods (Figure 2). The mean odds in the first period was 6.61 (a proportion of 87%), and the mean OR period effect between the second and first period was 0.32, which was equivalent to an odds of 2.12 (proportion of 68%) in the second period. The covariance matrices for each of the four scenarios are given in Data S1 and are described in the succeeding texts:

1. *Common period effect*, *high variability*:
  The period effect was common to all clusters with between‐cluster variance = 1.81. This was the amount of between‐cluster variability observed in year 1 of the motivating example. This represents a simple scenario with a large intracluster correlation coefficient (ICC = 0.20), where the standard model would have a correctly specified period effect.
2. *Common period effect*, *low variability*:
  The period effect was common to all clusters with between‐cluster variance = 0.25. This was the amount of between‐cluster variability observed in year 2 of the motivating example. This represents a simple scenario with a lower ICC (ICC = 0.05), where, again, the standard model would have a correctly specified period effect.
3. *Varying period effect*, *decreasing variability*:
  The period effect varied between clusters with the variability between the clusters decreasing from the first period to the second period. The initial between‐cluster variance was 1.81, and the period effect variance was 1.89. The decrease in variability from period 1 to period 2 resulted from a negative covariance between the initial value and the period effect of −1.72. This complex scenario reflects the underlying trends seen in the motivating example. In this scenario, the standard model would have a misspecified period effect.
4. *Varying period effect*, *stable variability*:
  The period effect varied between the clusters, but the between‐cluster variance remained the same for both periods. Here, the initial between‐cluster variability and period effect variability remained the same as in scenario (3), but the covariance was reduced to −0.94. This scenario was chosen to assess the effect of a varying period effect without the additional complication of the between‐cluster variation reducing in the second period. In this scenario, the standard model would have a misspecified period effect.

We simulated two scenarios for the intervention effect; these were not based on the motivating example:

- An intervention effect that was common to all clusters. We simulated an intervention effect log(OR) = 0.41 (equivalent to OR = 1.5) for all clusters. We also simulated log(OR) = 0 to calculate the type I error rate. In these scenarios, the standard model would have a correctly specified intervention effect.
- An intervention effect that varied between clusters drawn from the distribution log(OR) ~ *N*(0.41, 0.3). This gave a geometric mean OR = 1.5 with an IQR = 1.05–1.97. We also simulated a distribution log(OR) ~ *N*(0, 0.3) to calculate the type I error rate. In these scenarios, the standard model would have a misspecified intervention effect.

The variation in the intervention effect was modelled as being independent of the underlying outcome and period effect between‐cluster variability. This meant that the intervention effect varying between clusters would lead to increased variability between the clusters in period 2 as more clusters were receiving the intervention in this period.

Each scenario led to the odds of the outcome occurring in each cluster‐period. From this, the observations within each cluster‐period were sampled from a binomial distribution, assuming independence within each cluster‐period. This assumes a cross‐sectional design and is a deviation from the motivating example, where children were observed multiple times during the study, chosen for simplicity.

All combinations of these parameters were simulated.

### 3.2 Simulation study 2

Second, we hypothesised that the horizontal comparisons would depend on the model assumptions more heavily than the vertical comparisons. To aid interpretation of the results of simulation study 1, we sought to investigate the contribution of the horizontal comparisons to each analysis in each scenario.

In the trial design used for this paper, only group 2 contributed horizontal comparisons because groups 1 and 3 remained in the same condition for both periods of the study (Figure 1). This meant that we could investigate the weights given to the horizontal and vertical comparisons by identifying how much weight was given to group 2 relative to groups 1 and 3.

To do this, we reran the simulations but with an intervention effect log(OR) = 1.5 in group 2 of the trial but kept an intervention effect in group 1 of log(OR) = 0.41. An unbiased intervention effect estimate from horizontal comparisons alone would have an expectation of *E*(*l*og(OR)) = 1.5. An unbiased intervention effect estimate from vertical comparisons alone would have an expectation of 0.41 < *E*(log(OR)) < 1.5 depending on the weights given to each cluster and to periods 1 and 2 of the trial. Comparing the intervention effect estimates of each model in each scenario to the horizontal comparison *E*(log(OR)) = 1.5 allowed us to see how much the horizontal comparisons contributed to the analysis compared with the vertical comparisons. Such a large imbalance in the intervention effect between groups is, of course, unlikely (although not impossible); this simulation study was designed to investigate the contributions of vertical and horizontal comparisons, rather than to explore a realistic scenario.

## 4 Analysis models

Each simulated data set was analysed with three analysis models, each making different assumptions about the period effect and intervention effect.

### 4.1 Standard model

First, we used the standard method of analysis 4, 5: a mixed‐effect logistic regression with a random intercept and fixed effects for intervention effect and period effect:

(1) $$y_{\mathrm{ijk}}=\mu +\beta Z_{j}+\theta X_{\mathrm{ij}}+u_{i}\;$$

where *y*_ijk_ is the log odds of the outcome in cluster *i* in year *j* for observation *k*, μ is the mean log odds of the outcome in period 1 in the control condition, *β* is the period effect log‐OR comparing the outcome in periods 2 and 1, *Z*_j_ is an indicator of year, 0 for the first year and 1 for the second year, *θ* is the intervention effect log‐OR, and *X*_ij_ is an indicator of whether cluster *i* received the intervention in year *j*, $u_{i}\sim N\left(0,\sigma_{u}^{2}\right)$ is a random intercept allowing for variability in the outcome between clusters.

This model assumes that the period effect and the intervention effect are common to all clusters so is a misspecified model in scenarios where either the period effect or intervention effect varied between clusters.

### 4.2 Random period model

Second, we added a random effect for period to the standard model:

(2) $$y_{\mathrm{ijk}}=\mu +\left(\beta +v_{i}\right)Z_{j}+\theta X_{\mathrm{ij}}+u_{i}$$

where $\left(\begin{array}{c} u_{i}\\ v_{i} \end{array}\right)\sim \mathrm{MVN}\left(\left(\begin{array}{c} 0\\ 0 \end{array}\right),\left(\begin{array}{cc} \sigma_{u}^{2} & \sigma_{u,v\;}^{2}\\ \sigma_{u,v\;}^{2} & \sigma_{v}^{2} \end{array}\right)\right)$ are a random intercept and random effect for period respectively.

This model assumes that the intervention effect is common to all clusters but allows the period effect to vary between clusters. It is a misspecified model in scenarios where the intervention effect varies between the clusters.

Sometimes, other literatures have used a different model to allow the period effect to vary between the clusters 11, 12. For details on how these models relate to one another, see Data S2.

### 4.3 Random intervention model

Third, we added a random effect for the intervention to the standard model:

(3) $$y_{\mathrm{ijk}}=\mu +\beta Z_{j}+\left(\theta +z_{i}\right)X_{\mathrm{ij}}+u_{i}$$

where $\left(\begin{array}{c} u_{i}\\ z_{i} \end{array}\right)\sim \mathrm{MVN}\left(\left(\begin{array}{c} 0\\ 0 \end{array}\right),\left(\begin{array}{cc} \sigma_{u}^{2} & \sigma_{u,z\;}^{2}\\ \sigma_{u,z\;}^{2} & \sigma_{z}^{2} \end{array}\right)\right)$ are a random intercept and random effect for intervention respectively.

This model assumes that the period effect is common to all clusters but allows the intervention effect to vary between clusters. The model is a misspecified model in scenarios where the period effect varies between the clusters.

Whilst the random period and random intervention models allow for variability in the period and intervention effect respectively, they can estimate a variability of close to zero if the effect is common to all clusters. The random period model is correctly specified in the scenario with common period effect, and likewise, the random intervention model is correctly specified in the scenario with common intervention effect. Similarly, the random intervention model allows for a covariance between the intervention effect and the intercept ( $\sigma_{u,z\;}^{2}$) but allows this covariance to be zero, as is the case in our simulation study.

## 5 Estimands and performance measures

We ran 500 simulations for each combination of parameters. This allowed us to estimate the intervention effect to within 5% accuracy, assuming a variance estimate of 0.05. This variance is conservative as it is larger than the estimated variance we saw in the motivating example.

From the analysis models, we collected the estimated fixed effects, their standard errors, and the estimated between‐cluster covariance matrix.

We calculated the mean, standard deviations, 95% confidence intervals (CIs), and the IQR of the intercept, intervention effect, and period effect estimates from the 500 simulations. We calculated percentage bias as

$$\text{percentage bias}=\left(\frac{\bar{\hat{\beta }}-\beta }{\beta }\right)$$

where *β* is the true effect and $\bar{\hat{\beta }}$ is the mean of the effect estimates.

We calculated the coverage of 95% CIs as the proportion of simulations with the true effect contained within the 95% CI of the estimate. We calculated the type 1 error rate as the proportion of simulations with true OR = 1 with *P* < 0.05 against a null of the intervention effect OR = 1.

In the set of simulations with a different intervention effect in group 2 (simulation study 2), we compared the mean of the intervention effect estimates with the horizontal intervention effect of log(OR) = 1.5.

Simulations were run in r version 3.2; the lme4 package was used for mixed‐effect models.

## 6 Results

### 6.1 Model convergence

The standard model converged in all simulations for both simulation studies. When either the period effect or the intervention effect varied between clusters, the random period and random intervention models also converged in >99% of all simulations. However, when both period effect and intervention effect were common to all clusters, the random period model failed to converge in 3% to 9% of simulations and the random intervention model failed to converge in 4% to 33% of simulations. Estimates from these models were excluded from performance statistics. Further details of convergence of the models are given in Data S3.

### 6.2 Simulation study 1 results

#### 6.2.1 Bias of fixed‐effect estimates

**Figure 3:**
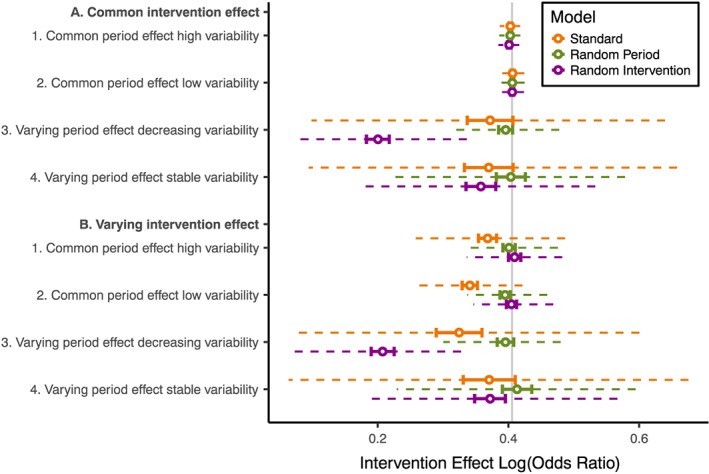
Comparison of intervention effect log(OR) from different analysis models and scenarios with true geometric mean intervention effect log(OR) = 0.41 in all groups. Vertical grey line: true log(OR). Hollow point: mean estimate. Solid barred line: 95% confidence interval. Dashed line: interquartile range (IQR) of estimates.

Figure 3 gives the mean and IQR of intervention effect estimates for each scenario. A table of the mean values is given in Data S4.

Where there were common period and intervention effects, all three models performed similarly, with estimation of the intervention effect in line with the true underlying effect.

Where the period effect varied between the clusters, only the random period model gave unbiased estimates of the intervention effect. Depending on the scenario, the standard model had between −20% and −8% bias and the random intervention model between −51% and −8% bias. Bias was larger when the period effect varied with decreasing variability than with stable variability but was similar regardless of whether there was a common or varying intervention effect. We also observed bias in the period effect estimates and intercept estimates from the standard model and random intervention model (Data S5 and S6).

Where the intervention effect varied between the clusters and there was a common period effect, the random intervention model and the random period model gave unbiased estimates of the intervention effect. Only the standard model intervention effect estimates had substantial bias (−9% and −16% bias for common period effects with high and low variabilities respectively).

Where either the period effect or intervention effect varied between clusters, the standard model intervention effect estimates had greater variability compared with the random period model or random intervention model. Differences were larger when the period effect varied between clusters than when the intervention effect varied between clusters. For example, the standard model intervention effect estimates were 3.6 times as variable as the random period model estimates when the period effect varied between clusters with decreasing variability with common intervention effect, whereas the standard model intervention effect estimates were 1.5 times as variable as the random intervention model estimates when the intervention effect varied between clusters with common period effect with high variability.

#### 6.2.2 Standard errors, coverage, and type 1 error

**Figure 4:**
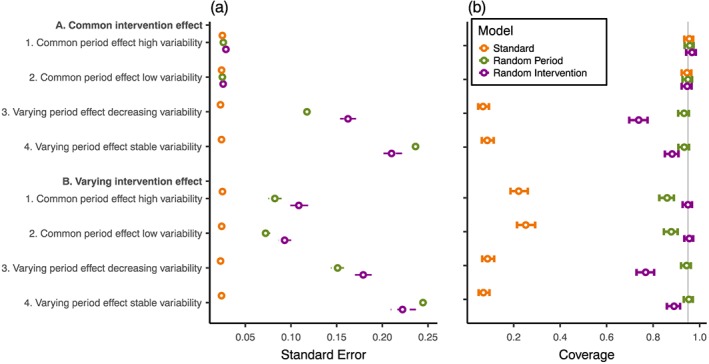
Comparison of estimated intervention effect (a) standard errors and (b) 95% confidence interval coverage for different analysis models and scenarios with a geometric mean intervention effect of log(OR) = 0.41 in all groups. Vertical grey line: 95% coverage. Hollow point: mean estimate. Solid barred line: 95% confidence interval. Dashed line: interquartile range (IQR) of estimates.

In scenarios with a common period and intervention effect, 95% coverage was maintained regardless of the analysis model and the estimated standard errors were similar across analysis models (Figure 4 and Data S7 and S8).

When period effect or intervention effect varied between clusters, the standard model gave standard errors that were markedly smaller than the random period model and random intervention model. The mean intervention effect standard error from the standard model was less than 0.33 and 0.26 times the mean standard error of the random period model and random intervention model respectively.

The inappropriately small standard errors given by the standard model were in part explained by downward bias in the estimation of between‐cluster variability (Data S9). For example, when variability was stable over the two time periods with a variance of 1.79, the standard model estimated the variance as 1.26.

The bias in estimates, standard errors, and increased variability in estimates led to undercoverage of the 95% CIs of the intervention effect estimates (Figure 4). For the standard model, undercoverage was severe when either the intervention effect or the period effect varied between clusters (<25% coverage). Similarly, the random intervention model had undercoverage when the period effect varied between clusters (74% and 88% coverage for decreasing and stable variability respectively) regardless of intervention effect variability. Finally, the random period model had undercoverage of CIs when the intervention effect varied between clusters with a common period effect (86% and 88% coverage for common period effect with high and low variabilities respectively).

Type 1 error rates followed the same patterns as coverage (Data S10).

### 6.3 Simulation study 2 results

**Figure 5:**
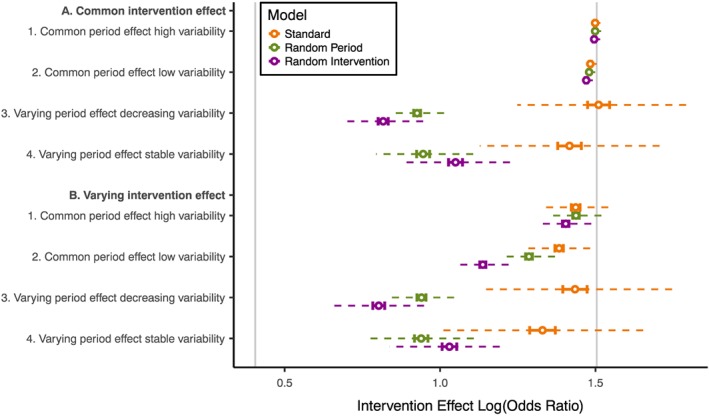
Comparison of intervention effect log odds ratios from different analysis models for all scenarios with the intervention effect larger in group 2 than group 1. Vertical grey lines: true intervention effect in group 1 (log(OR) = 0.41) and group 2 (log(OR) = 1.5). Hollow point: mean estimate. Solid barred line: 95% confidence interval. Dashed line: interquartile range (IQR) of estimates.

Figure 5 gives the estimated log(OR) for each scenario where the group 1 and 2 intervention effects differed (log(OR) = 0.41 in group 1 and log(OR) = 1.5 in group 2).

All analysis models gave a mean estimated intervention effect close to the group 2 effect when there was a common period effect and a common intervention effect; this was the case in the high and low variability scenarios. This suggests that, in these scenarios, the intervention effect is largely estimated from horizontal within‐cluster comparisons in group 2; groups 1 and 3 appeared to contribute to estimation of the period effect but had little influence on the intervention effect estimate.

The standard model estimates remained close to the group 2 intervention effect in all scenarios. The downward bias we observed in our first set of simulations suggests that at least some of the movement away from the group 2 effect is because of bias and not because of a reduction in the contribution of the horizontal comparisons. This implies that the standard model was continuing to estimate the intervention effect largely from horizontal comparisons in group 2.

In contrast, when the period effect varied between clusters, the random period model gave intervention effect estimates much further from the horizontal comparison estimates. This implies that the horizontal comparisons in group 2 could not contribute as much information to the analysis because there was less certainty about separating the period effect and intervention effect in these comparisons. This was similar in the scenarios where the intervention effect varied between clusters but the period effect was common for both the random period and random intervention models, but to a smaller degree.

## 7 Example

For our motivating example, we hypothesised that the standard model gave a larger intervention effect than either of the two time periods analysed separately because the standard model was misspecified by ignoring variability in either the period effect or the intervention effect. Our simulation study suggests that this is not the case because we would expect the standard model to underestimate the intervention effect with these types of misspecification, rather than overestimate the effect. However, we also found that the standard model gave a very large weight to the horizontal comparisons. This does help to explain the counterintuitive results seen in the motivating example 2.

We reanalysed the deworming trial by using the three analysis models investigated in the simulation study and additionally looked at years 1 and 2 separately by using a mixed‐effect model with a fixed effect for intervention and a random intercept to attain estimates for the intervention effect from vertical comparisons. In line with the published reanalysis of this study, we ignored pupil‐level clusters from multiple observations of the same pupils; this is in line with research suggesting that it is sufficient to adjust for the highest level of clustering alone, known as passing the buck 13.

The results in Table 2 are different from the published reanalysis as we have used a different version of the data (see Data S11 for details) and have not adjusted for covariates other than period 14.

**Table 2:** Intervention effect estimates from motivating example with different analysis models.

Model	Odds ratio (95% CI)	Standard error	*P*‐value	*P*‐value of random period or intervention effect
Separate year analysis (vertical comparisons)				
Year 1	1.67 (0.90,3.10)	0.32	0.11	
Year 2	1.19 (0.95, 1.50)	0.12	0.13	
Combined analysis				
Standard model	1.74 (1.67, 1.81)	0.02	<0.001	
Random period model	1.26 (1.02, 1.57)	0.11	0.03	<0.001
Random intervention model	1.25 (0.96, 1.62)	0.13	0.09	<0.001

We found that the standard model combining data from both years of the study gave a larger estimate of the intervention effect than either year analysed separately, which is as was found in the reanalysis 4.

Adjusting for variation between clusters in the period effect or intervention effect (i.e. using either the random period model or random intervention model) increased the intervention effect standard error and reduced the intervention effect towards the null. Both approaches gave an intervention effect estimate between the estimated effect in year 1 and year 2. This suggests that the horizontal comparisons are contributing less to these analysis models than to the standard model; this is consistent with the findings of our second simulation study into the contribution of the horizontal comparisons.

The random period model found strong evidence of variability in the period effect (*p* < 0.001), and the random intervention model found strong evidence of variability in the intervention effect (*p* < 0.001). Because the period effect and intervention effect are confounded with one another, evidence of variability in the intervention effect could be caused by variability in the period effect or vice versa. The random period model estimated a between‐cluster covariance matrix similar to the simulation study scenario with varying period effect with decreasing variability. The random intervention model estimated lower variability between clusters in the intervention condition than in the control condition because of the reduced variability in year 2. This is a scenario that we did not consider in our simulation study where we only investigated a scenario with greater variability in the intervention condition. Inspection of the data suggests that the random period model is the most appropriate one. A mixed‐effect model with a random effect for period run on observations from group 3, which never received the intervention, finds strong evidence of variability in the period effect (*p* < 0.001). But a mixed‐effect model with a random effect for intervention run on observations from groups 1 and 3, where the intervention effect is not confounded with the period effect, finds no evidence of variability in the intervention effect (*p* = 0.34).

The random period model suggests that there is some evidence that the deworming intervention increased school attendance (OR = 1.26, 95% CI 1.02, 1.57; *p* = 0.03). The effect found by using this model is weaker, both in terms of absolute size and level of statistical significance, than the effect found by using the standard model. There are still limitations in these data and this analysis, on which further information has been published elsewhere 1, 2, 3.

## 8 Discussion

We found biased estimates and serious undercoverage of CIs in the SWT scenarios we simulated when the analysis model ignored variability between clusters in the period effect or intervention effect. In these scenarios, results from the standard model were driven largely by the horizontal comparisons.

We have shown that, in the scenarios we considered, misspecifying the random effects of mixed‐effect models can result in biased intervention effect estimates. The standard model underestimated the intervention effect when either the period or the intervention effect varied between the clusters. The underestimation when the period effect varied may result from the standard model estimating an intervention effect averaged over the two periods, whereas the true effect for this scenario was a within‐period intervention effect. This is analogous to the difference between the population‐averaged effect and the cluster‐specific effects that are given by different analysis methods. In the presence of intervention effect variability, the standard model also gave biased estimates of the intervention effect. The random intervention model had even larger bias when it was misspecified than the standard model. Conversely, the random period model had only negligible bias in estimates in all scenarios we considered. These results are consistent with previous research into misspecifying mixed‐effect models in cluster randomised trials 7, 9. We have built on this literature and shown that these results extend to SWTs. This highlights how sensitive mixed‐effect models can be to misspecification of model assumptions.

Caution is needed beyond estimation of the intervention effect itself. In our simulation study, the bias extended to standard errors and between‐cluster variability. The latter has implications for reporting the ICC, as recommended by the Consolidated Standards of Reporting Trials guidelines 15. In addition to the implications for inference, the bias in standard errors has implications for determining the power and sample size of SWTs. Because the standard error from the standard model is used in most current methods of SWT sample size calculations 12, 16, 17, 18, they should not be applied when the period effect or intervention effect is expected to vary between clusters, at least in relation to the characteristics of the trial exemplar used in this paper. Instead, the method developed by Hooper *et al*. may be more appropriate 11.

The result of these biases was undercoverage of CIs for the intervention effect. If model assumptions do not hold, we risk being overconfident in our conclusions. We found particularly severe undercoverage when using the standard model. This has been seen in previous research into misspecified random effects 6, 19 and has recently been seen in the setting of SWTs 20. This is reflected in our analysis of the motivating example; we see a large increase in the standard error of the intervention effect, and so CIs are much wider when moving from the standard model to the random period model or random intervention model.

The results from our simulation study could be explained by the excessive weight given to the horizontal comparisons, even with a lower ICC = 0.05. Because the horizontal comparisons are within‐cluster comparisons, they avoid the additional variability of between‐cluster variation. This means that if the period and intervention effects can be separated, the horizontal comparisons will be given more weight than the vertical comparisons by all the analysis models we considered. However, by making the stringent assumption that period and intervention effects are the same in every cluster, the standard model assumes too much certainty in separating the period and intervention effects. The reason that the standard model performed poorly in the simulation study was because of its reliance on the horizontal comparisons.

In the design we studied, the weight given to horizontal comparisons also meant that greater weight was given to some groups of clusters than others. The implications of this are not well understood. When there is a large difference in the weight given to each group, the intervention effect estimate no longer represents an average effect across the clusters and interpretation becomes more difficult. Further research is needed to explore this issue in more traditional SWT designs with more groups and when all clusters have observations in the control and intervention conditions, and so all clusters contribute through horizontal comparisons.

A criticism of the random intervention model and, to a lesser extent, the random period model is that they sometimes had problems with convergence. This occurred almost exclusively when both the period effect and the intervention effect were common to all clusters; the non‐convergence resulted from the models attempting to estimate a true variance of zero, the boundary of the parameter. In this scenario, all the analysis models gave unbiased effect estimates and appropriate CI coverage. We would suggest that an analysis plan gives an alternative, simpler model to use in case of convergence issues due to lack of variability. In our simulation study, this procedure gave good coverage and no bias in the scenarios with common period effect and intervention effect, where convergence was an issue (data not shown).

Given that the mixed‐effect model can be so sensitive to model assumptions, other analysis methods should be considered. This choice should be prespecified and prior knowledge used to justify the assumptions made by the chosen analysis method. We found the random period model to be the most robust of the models considered, but there was still undercoverage of CIs in some scenarios. Some have suggested using permutation tests on the standard model 20. Although this will give correct inference, there is still a risk of biased intervention effect estimation. Alternative analysis methods that make fewer assumptions may be more appropriate. Generalised estimating equations have been suggested for the analysis of SWTs 21 and have been shown to be more robust to misspecification of the correlations in the data in other settings 22, but this robustness has yet to be assessed in the context of SWTs. Analysis methods that only make use of the vertical comparisons are desirable as they require no assumptions about period effects, but there are no such methods currently published, and these analyses are less efficient 23. Sensitivity analysis could also be used to assess the robustness of results.

We have only considered a limited range of designs in this simulation study. We used a very simple SWT design to make the analyses as transparent as possible; this design only had two steps, and not all clusters received the intervention in the course of the study. Further research is needed to confirm that our findings hold for other SWT designs. In more traditional SWTs, all clusters receive both the control and intervention conditions, and so all clusters contribute horizontal comparisons. Because the problems we highlight arise from the horizontal comparisons, this might exacerbate the problems we identified. We have only considered two values for the ICC when the period effect was common to all clusters and have not assessed the effect of ICC when period effects vary between clusters. In scenarios where these effects varied between clusters, the baseline ICC was 0.20, which, in many contexts, would be considered large. Additionally, there was large variability in the period effect; the effect of a less variable period effect needs further exploration. It is not known how common it is for the period and intervention effects to vary between clusters in practice; however, we have based this simulation on real trial data. Large clusters were used in the simulation study to reflect the motivating deworming trial; however, similar results were seen with a smaller mean cluster size of 250 (data not shown). We used a large number of clusters in each group to avoid small sample issues.

Whilst further research is needed to explore the potential for bias in a wider range of designs and settings, we have demonstrated that there is a potential for the standard model to give biased intervention effect estimates and undercoverage of CIs. These simulations provide clear evidence that the standard model for analysis of SWTs can be both highly sensitive to the data meeting the model assumptions and highly dependent on non‐randomised horizontal comparisons. We urge those conducting SWTs to ensure an appropriate analysis is used.

## Data Accessibility

## Supporting information

Data S1: Covariance matrices
Data S2: Model parameterisation
Data S3: Table of convergence of analysis models by simulation parameters
Data S4: Table of mean intervention effect log odds ratio estimates from simulations
Data S5a: Figure of estimated intercepts
Data S5b: Table of mean intercept log odds estimates from simulations
Data S6a: Figure of estimated Period effects
Data S6b: Table of mean period effect log odds ratio estimates from simulations
Data S7: Table of mean standard error estimates from simulations
Data S8: Table of coverage of 95% confidence intervals
Data S9a: Estimation of intercept between‐cluster variance
Data S9b: Table of mean intercept variance (between‐cluster variance) estimates from simulations
Data S10a: Figure of Type 1 error
Data S10b: Table of Type 1 error rate of simulations
Data S11: Deworming trial data cleaning
