# Supplementary material for: Bias and inference from misspecified mixed‐effect models in stepped wedge trial analysis
Source: Stat Med. 2017 May 28;36(23):3670–82. doi: 10.1002/sim.7348 (PMC5600088; doi:10.1002/sim.7348)
Supplement: Supplementary file 1 — Data S1: Covariance matrices Data S2: Model parameterisation Data S3: Table of convergence of analysis models by simulation parameters Data S4: Table of mean intervention effect log odds ratio estimates from simulations Data S5a: Figure of estimated intercepts Data S5b: Table of mean intercept log odds estimates from simulations Data S6a: Figure of estimated Period effects Data S6b: Table of mean period effect log odds ratio estimates from simulations Data S7: Table of mean standard error estimates from simulations Data S8: Table of coverage of 95% confidence intervals Data S9a: Estimation of intercept between‐cluster variance Data S9b: Table of mean intercept variance (between‐cluster variance) estimates from simulations Data S10a: Figure of Type 1 error Data S10b: Table of Type 1 error rate of simulations Data S11: Deworming trial data cleaning [file SIM-36-3670-s001.docx]

**Supplementary Information**

**S1: Covariance matrices**

Below are the covariance matrices used in the data generating process for each scenario. These are given in the format:

$$\left( \begin{matrix} var(intercept) & cov(period, intercept) \\ cov(period, intercept) & var(period) \end{matrix} \right)$$

1. *Common period effect, high variability*

$$\left( \begin{matrix} 1.808 & 0 \\ 0 & 0 \end{matrix} \right)$$

2. *Common period effect, low variability*

$$\left( \begin{matrix} 0.251 & 0 \\ 0 & 0 \end{matrix} \right)$$

3. *Varying period effect, decreasing variability*

$$\left( \begin{matrix} 1.808 & -1.721 \\ -1.721 & 1.885 \end{matrix} \right)$$

4. *Varying period effect, stable variability*

$$\left( \begin{matrix} 1.808 & -0.943 \\ -0.943 & 1.885 \end{matrix} \right)$$

**S2: Model parameterisation**

In this paper, we used the following model to allow the period effect to vary between clusters:

*Parameterisation A*

$$y_{ijk}=\mu+\left( \beta+v_{i} \right)Z_{j} +\theta X_{ij} + u_{i}$$

where $y_{ijk}$ is the log odds of the outcome in cluster *i* in year *j* for observation *k*, $\mu$ is the mean log odds of the outcome in period one in the control condition,$\beta$ is the period effect log odds ratio comparing the outcome in periods two and one, $Z_{j}$is an indicator of year; 0 for the first year and 1 for the second year, $\theta$ is the intervention effect log odds ratio, and $X_{ij}$ is an indicator of whether cluster *i* received the intervention in year *j*, and $\left( \begin{matrix} u_{i} \\ v_{i} \end{matrix} \right)\sim MVN\left( \left( \begin{matrix} 0 \\ 0 \end{matrix} \right),\left( \begin{matrix} \sigma_{u}^{2} & \sigma_{u,v}^{2} \\ \sigma_{u,v}^{2} & \sigma_{v}^{2} \end{matrix} \right) \right)$ are a random intercept and random effect for period respectively.

Other literature has sometimes used an alternative parameterisation:

*Parameterisation B*

$$y_{ijk}=\mu+\beta Z_{j} +\theta X_{ij} +v_{ij}^{*}+ u_{i}^{*}$$

Where now $u_{i}^{*}\sim N(0,\sigma_{u^{*}}^{2})$ and $v_{ij}^{*}\sim N(0,\sigma_{v^{*}}^{2})$.

The parameterisation used in this paper, parameterisation A, is more flexible than the parameterisation sometimes used elsewhere, parameterisation B. Parameterisation A allows the total variability to change between periods. This is necessary to correctly model our motivating example. Parameterisation B assumes that the total variability is the same in each period.

We can add the restraint that the variance is the same in each period to parameterisation A by setting $\sigma_{u,v}^{2}=-\frac{1}{2}\sigma_{v}^{2}$. When we do this, parameterisations A and B are equivalent and

$$\sigma_{u^{*}}^{2}=\sigma_{u}^{2}-\frac{1}{2}\sigma_{v}^{2}$$

$$\sigma_{v^{*}}^{2}=\frac{1}{2}\sigma_{v}^{2}$$

**S3: Table of convergence of analysis models by simulation parameters**

| **Group two Intervention effect log(OR)** | **Group one Intervention effect log(OR) ^a^** | **Intervention effect** | **Period effect** | **Model convergence, %** | | |
| --- | --- | --- | --- | --- | --- | --- |
|  |  |  |  | **Standard**  **Model** | **Random**  **Period Model** | **Random Intervention Model** |
| Same as  group 1 log(OR)=0.41 | 0.41 | Common | Common period effect, high variability | 100 | 95 | 67 |
|  | 0.41 | Common | Common period effect, low variability | 100 | 96 | 94 |
|  | 0.41 | Common | Varying period effect, Decreasing variability | 100 | 99 | 100 |
|  | 0.41 | Common | Varying period effect, Stable variability | 100 | 100 | 100 |
|  | 0.41 | Varying | Common period effect, high variability | 100 | 100 | 100 |
|  | 0.41 | Varying | Common period effect, low variability | 100 | 100 | 100 |
|  | 0.41 | Varying | Varying period effect, Decreasing variability | 100 | 100 | 100 |
|  | 0.41 | Varying | Varying period effect, Stable variability | 100 | 100 | 100 |
|  | 0 | Common | Common period effect, high variability | 100 | 95 | 73 |
|  | 0 | Common | Common period effect, low variability | 100 | 97 | 96 |
|  | 0 | Common | Varying period effect, Decreasing variability | 100 | 100 | 100 |
|  | 0 | Common | Varying period effect, Stable variability | 100 | 100 | 100 |
|  | 0 | Varying | Common period effect, high variability | 100 | 100 | 100 |
|  | 0 | Varying | Common period effect, low variability | 100 | 100 | 100 |
|  | 0 | Varying | Varying period effect, Decreasing variability | 100 | 100 | 100 |
|  | 0 | Varying | Varying period effect, Stable variability | 100 | 100 | 100 |
| Different to group 1 log(OR)=1.5 | 0.41 | Common | Common period effect, high variability | 100 | 91 | 73 |
|  | 0.41 | Common | Common period effect, low variability | 100 | 99 | 94 |
|  | 0.41 | Common | Varying period effect, Decreasing variability | 100 | 100 | 100 |
|  | 0.41 | Common | Varying period effect, Stable variability | 100 | 100 | 100 |
|  | 0.41 | Varying | Common period effect, high variability | 100 | 100 | 100 |
|  | 0.41 | Varying | Common period effect, low variability | 100 | 100 | 100 |
|  | 0.41 | Varying | Varying period effect, Decreasing variability | 100 | 100 | 100 |
|  | 0.41 | Varying | Varying period effect, Stable variability | 100 | 100 | 100 |

^a^ In scenarios where this effect varies between clusters this is the geometric mean effect

**S4: Table of mean intervention effect log odds ratio estimates from simulations**

| **Group two Intervention effect log(OR)** | **Group one Intervention effect log(OR) ^a^** | **Intervention effect** | **Period effect** | **Mean log odds ratio** | | |
| --- | --- | --- | --- | --- | --- | --- |
|  |  |  |  | **Standard**  **Model** | **Random**  **Period Model** | **Random Intervention Model** |
| Same as  group 1 log(OR)=0.41 | 0.41 | Common | Common period effect, high variability | 0.40 | 0.40 | 0.40 |
|  | 0.41 | Common | Common period effect, low variability | 0.41 | 0.41 | 0.41 |
|  | 0.41 | Common | Varying period effect, Decreasing variability | 0.37 | 0.40 | 0.20 |
|  | 0.41 | Common | Varying period effect, Stable variability | 0.37 | 0.40 | 0.36 |
|  | 0.41 | Varying | Common period effect, high variability | 0.37 | 0.40 | 0.41 |
|  | 0.41 | Varying | Common period effect, low variability | 0.34 | 0.39 | 0.40 |
|  | 0.41 | Varying | Varying period effect, Decreasing variability | 0.32 | 0.40 | 0.21 |
|  | 0.41 | Varying | Varying period effect, Stable variability | 0.37 | 0.41 | 0.37 |
|  | 0 | Common | Common period effect, high variability | 0 | 0 | 0 |
|  | 0 | Common | Common period effect, low variability | 0 | 0 | 0 |
|  | 0 | Common | Varying period effect, Decreasing variability | -0.01 | -0.01 | -0.18 |
|  | 0 | Common | Varying period effect, Stable variability | -0.01 | -0.01 | -0.05 |
|  | 0 | Varying | Common period effect, high variability | -0.03 | 0 | 0 |
|  | 0 | Varying | Common period effect, low variability | -0.07 | -0.01 | -0.01 |
|  | 0 | Varying | Varying period effect, Decreasing variability | -0.07 | 0 | -0.18 |
|  | 0 | Varying | Varying period effect, Stable variability | -0.06 | -0.02 | -0.07 |
| Different to group 1 log(OR)=1.5 | 0.41 | Common | Common period effect, high variability | 1.50 | 1.50 | 1.50 |
|  | 0.41 | Common | Common period effect, low variability | 1.48 | 1.48 | 1.47 |
|  | 0.41 | Common | Varying period effect, Decreasing variability | 1.51 | 0.93 | 0.82 |
|  | 0.41 | Common | Varying period effect, Stable variability | 1.42 | 0.95 | 1.05 |
|  | 0.41 | Varying | Common period effect, high variability | 1.44 | 1.44 | 1.40 |
|  | 0.41 | Varying | Common period effect, low variability | 1.38 | 1.29 | 1.14 |
|  | 0.41 | Varying | Varying period effect, Decreasing variability | 1.43 | 0.94 | 0.80 |
|  | 0.41 | Varying | Varying period effect, Stable variability | 1.33 | 0.94 | 1.03 |

^a^ In scenarios where this effect varies between clusters this is the geometric mean effect

**S5a: Figure of estimated intercepts**

Comparison of intercept log odds from different analysis models for all scenarios with the same intervention effect in all groups. Hollow point: Mean estimate, solid barred line: 95% confidence interval, dashed line: IQR of estimates.

4. Varying period effect stable variability

3. Varying period effect decreasing variability

2. Common period effect low variability

1. Common period effect high variability

**B. Varying intervention effect**

4. Varying period effect stable variability

3. Varying period effect decreasing variability

2. Common period effect low variability

1. Common period effect high variability

**A. Common intervention effect**

1.5

1.6

1.7

1.8

1.9

Intercept Log(oods)

Model

Standard

Random Period

Random Intervention

**S5b: Table of mean intercept log odds estimates from simulations**

| **Group two Intervention effect log(OR)** | **Group one Intervention effect log(OR) ^a^** | **Intervention effect** | **Period effect** | **Mean log odds** | | |
| --- | --- | --- | --- | --- | --- | --- |
|  |  |  |  | **Standard**  **Model** | **Random**  **Period Model** | **Random Intervention Model** |
| Same as  group 1 log(OR)=0.41 | 0.41 | Common | Common period effect, high variability | 1.89 | 1.89 | 1.89 |
|  | 0.41 | Common | Common period effect, low variability | 1.89 | 1.89 | 1.89 |
|  | 0.41 | Common | Varying period effect, Decreasing variability | 1.55 | 1.89 | 1.71 |
|  | 0.41 | Common | Varying period effect, Stable variability | 1.78 | 1.89 | 1.82 |
|  | 0.41 | Varying | Common period effect, high variability | 1.90 | 1.89 | 1.89 |
|  | 0.41 | Varying | Common period effect, low variability | 1.92 | 1.89 | 1.89 |
|  | 0.41 | Varying | Varying period effect, Decreasing variability | 1.58 | 1.89 | 1.72 |
|  | 0.41 | Varying | Varying period effect, Stable variability | 1.78 | 1.89 | 1.82 |
|  | 0 | Common | Common period effect, high variability | 1.89 | 1.89 | 1.89 |
|  | 0 | Common | Common period effect, low variability | 1.89 | 1.89 | 1.89 |
|  | 0 | Common | Varying period effect, Decreasing variability | 1.55 | 1.89 | 1.72 |
|  | 0 | Common | Varying period effect, Stable variability | 1.77 | 1.89 | 1.83 |
|  | 0 | Varying | Common period effect, high variability | 1.90 | 1.89 | 1.89 |
|  | 0 | Varying | Common period effect, low variability | 1.92 | 1.89 | 1.89 |
|  | 0 | Varying | Varying period effect, Decreasing variability | 1.59 | 1.89 | 1.72 |
|  | 0 | Varying | Varying period effect, Stable variability | 1.80 | 1.89 | 1.83 |
| Different to group 1 log(OR)=1.5 | 0.41 | Common | Common period effect, high variability | 1.52 | 1.53 | 1.53 |
|  | 0.41 | Common | Common period effect, low variability | 1.53 | 1.53 | 1.54 |
|  | 0.41 | Common | Varying period effect, Decreasing variability | 1.19 | 1.72 | 1.64 |
|  | 0.41 | Common | Varying period effect, Stable variability | 1.43 | 1.71 | 1.71 |
|  | 0.41 | Varying | Common period effect, high variability | 1.55 | 1.54 | 1.59 |
|  | 0.41 | Varying | Common period effect, low variability | 1.57 | 1.60 | 1.77 |
|  | 0.41 | Varying | Varying period effect, Decreasing variability | 1.22 | 1.71 | 1.66 |
|  | 0.41 | Varying | Varying period effect, Stable variability | 1.46 | 1.71 | 1.72 |

^a^ In scenarios where this effect varies between clusters this is the geometric mean effect

True intercept is log(6.62)=1.89

**S6a: Figure of estimated Period effects**

Comparison of period effect log odds ratio from different analysis models for all scenarios with the same intervention effect in all groups. Hollow point: Mean estimate, solid barred line: 95% confidence interval, dashed line: IQR of estimates.

4. Varying period effect stable variability

3. Varying period effect decreasing variability

2. Common period effect low variability

1. Common period effect high variability

**B. Varying intervention effect**

4. Varying period effect stable variability

3. Varying period effect decreasing variability

2. Common period effect low variability

1. Common period effect high variability

**A. Common intervention effect**

-1.2

-1.0

-0.8

Period Effect Log(Odds Ratio)

Model

Standard

Random Period

Random Intervention

**S6b: Table of mean period effect log odds ratio estimates from simulations**

| **Group two Intervention effect log(OR)** | **Group one Intervention effect log(OR) ^a^** | **Intervention effect** | **Period effect** | **Mean log odds ratio** | | |
| --- | --- | --- | --- | --- | --- | --- |
|  |  |  |  | **Standard**  **Model** | **Random**  **Period Model** | **Random Intervention Model** |
| Same as  group 1 log(OR)=0.41 | 0.41 | Common | Common period effect, high variability | -1.13 | -1.13 | -1.13 |
|  | 0.41 | Common | Common period effect, low variability | -1.14 | -1.14 | -1.14 |
|  | 0.41 | Common | Varying period effect, Decreasing variability | -0.76 | -1.13 | -0.76 |
|  | 0.41 | Common | Varying period effect, Stable variability | -1.07 | -1.13 | -1.07 |
|  | 0.41 | Varying | Common period effect, high variability | -1.14 | -1.13 | -1.14 |
|  | 0.41 | Varying | Common period effect, low variability | -1.14 | -1.13 | -1.14 |
|  | 0.41 | Varying | Varying period effect, Decreasing variability | -0.77 | -1.13 | -0.77 |
|  | 0.41 | Varying | Varying period effect, Stable variability | -1.08 | -1.14 | -1.08 |
|  | 0 | Common | Common period effect, high variability | -1.14 | -1.14 | -1.14 |
|  | 0 | Common | Common period effect, low variability | -1.13 | -1.13 | -1.13 |
|  | 0 | Common | Varying period effect, Decreasing variability | -0.79 | -1.13 | -0.79 |
|  | 0 | Common | Varying period effect, Stable variability | -1.06 | -1.13 | -1.06 |
|  | 0 | Varying | Common period effect, high variability | -1.13 | -1.13 | -1.13 |
|  | 0 | Varying | Common period effect, low variability | -1.14 | -1.13 | -1.14 |
|  | 0 | Varying | Varying period effect, Decreasing variability | -0.79 | -1.14 | -0.79 |
|  | 0 | Varying | Varying period effect, Stable variability | -1.05 | -1.13 | -1.05 |
| Different to group 1 log(OR)=1.5 | 0.41 | Common | Common period effect, high variability | -1.13 | -1.13 | -1.13 |
|  | 0.41 | Common | Common period effect, low variability | -1.13 | -1.13 | -1.13 |
|  | 0.41 | Common | Varying period effect, Decreasing variability | -0.76 | -0.94 | -0.77 |
|  | 0.41 | Common | Varying period effect, Stable variability | -1.06 | -0.95 | -1.07 |
|  | 0.41 | Varying | Common period effect, high variability | -1.13 | -1.12 | -1.13 |
|  | 0.41 | Varying | Common period effect, low variability | -1.13 | -1.07 | -1.13 |
|  | 0.41 | Varying | Varying period effect, Decreasing variability | -0.76 | -0.95 | -0.76 |
|  | 0.41 | Varying | Varying period effect, Stable variability | -1.07 | -0.95 | -1.07 |

^a^ In scenarios where this effect varies between clusters this is the geometric mean effect

True period odds ratio is log(0.32) = -1.14

**S7: Table of mean standard error estimates from simulations**

| **Group two Intervention effect log(OR)** | **Group one Intervention effect log(OR) ^a^** | **Intervention effect** | **Period effect** | **Mean standard error** | | |
| --- | --- | --- | --- | --- | --- | --- |
|  |  |  |  | **Standard**  **Model** | **Random**  **Period Model** | **Random Intervention Model** |
| Same as  group 1 log(OR)=0.41 | 0.41 | Common | Common period effect, high variability | 0.02 | 0.03 | 0.03 |
|  | 0.41 | Common | Common period effect, low variability | 0.02 | 0.02 | 0.03 |
|  | 0.41 | Common | Varying period effect, Decreasing variability | 0.02 | 0.12 | 0.16 |
|  | 0.41 | Common | Varying period effect, Stable variability | 0.02 | 0.24 | 0.21 |
|  | 0.41 | Varying | Common period effect, high variability | 0.02 | 0.08 | 0.11 |
|  | 0.41 | Varying | Common period effect, low variability | 0.02 | 0.07 | 0.09 |
|  | 0.41 | Varying | Varying period effect, Decreasing variability | 0.02 | 0.15 | 0.18 |
|  | 0.41 | Varying | Varying period effect, Stable variability | 0.02 | 0.24 | 0.22 |
|  | 0 | Common | Common period effect, high variability | 0.02 | 0.03 | 0.03 |
|  | 0 | Common | Common period effect, low variability | 0.02 | 0.02 | 0.02 |
|  | 0 | Common | Varying period effect, Decreasing variability | 0.02 | 0.12 | 0.16 |
|  | 0 | Common | Varying period effect, Stable variability | 0.02 | 0.24 | 0.21 |
|  | 0 | Varying | Common period effect, high variability | 0.02 | 0.08 | 0.11 |
|  | 0 | Varying | Common period effect, low variability | 0.02 | 0.07 | 0.09 |
|  | 0 | Varying | Varying period effect, Decreasing variability | 0.02 | 0.15 | 0.18 |
|  | 0 | Varying | Varying period effect, Stable variability | 0.02 | 0.24 | 0.22 |
| Different to group 1 log(OR)=1.5 | 0.41 | Common | Common period effect, high variability | 0.03 | 0.03 | 0.03 |
|  | 0.41 | Common | Common period effect, low variability | 0.03 | 0.03 | 0.03 |
|  | 0.41 | Common | Varying period effect, Decreasing variability | 0.03 | 0.16 | 0.19 |
|  | 0.41 | Common | Varying period effect, Stable variability | 0.03 | 0.26 | 0.23 |
|  | 0.41 | Varying | Common period effect, high variability | 0.03 | 0.08 | 0.11 |
|  | 0.41 | Varying | Common period effect, low variability | 0.03 | 0.09 | 0.13 |
|  | 0.41 | Varying | Varying period effect, Decreasing variability | 0.03 | 0.19 | 0.20 |
|  | 0.41 | Varying | Varying period effect, Stable variability | 0.03 | 0.27 | 0.24 |

^a^ In scenarios where this effect varies between clusters this is the geometric mean effect

**S8: Table of coverage of 95% confidence intervals**

| **Group two Intervention effect log(OR)** | **Group one Intervention effect log(OR) ^a^** | **Intervention effect** | **Period effect** | **Coverage %** | | |
| --- | --- | --- | --- | --- | --- | --- |
|  |  |  |  | **Standard**  **Model** | **Random**  **Period Model** | **Random Intervention Model** |
| Same as  group 1 log(OR)=0.41 | 0.41 | Common | Common period effect, high variability | 96 | 96 | 97 |
|  | 0.41 | Common | Common period effect, low variability | 95 | 95 | 95 |
|  | 0.41 | Common | Varying period effect, Decreasing variability | 7 | 93 | 74 |
|  | 0.41 | Common | Varying period effect, Stable variability | 9 | 93 | 88 |
|  | 0.41 | Varying | Common period effect, high variability | 22 | 86 | 95 |
|  | 0.41 | Varying | Common period effect, low variability | 25 | 88 | 96 |
|  | 0.41 | Varying | Varying period effect, Decreasing variability | 9 | 94 | 77 |
|  | 0.41 | Varying | Varying period effect, Stable variability | 7 | 95 | 89 |
|  | 0 | Common | Common period effect, high variability | 96 | 97 | 96 |
|  | 0 | Common | Common period effect, low variability | 95 | 95 | 95 |
|  | 0 | Common | Varying period effect, Decreasing variability | 9 | 96 | 80 |
|  | 0 | Common | Varying period effect, Stable variability | 8 | 95 | 88 |
|  | 0 | Varying | Common period effect, high variability | 22 | 85 | 95 |
|  | 0 | Varying | Common period effect, low variability | 26 | 83 | 93 |
|  | 0 | Varying | Varying period effect, Decreasing variability | 10 | 95 | 82 |
|  | 0 | Varying | Varying period effect, Stable variability | 9 | 95 | 88 |
| Different to group 1 log(OR)=1.5 | 0.41 | Common | Common period effect, high variability | 0 ^b^ | 0 ^b^ | 0 ^b^ |
|  | 0.41 | Common | Common period effect, low variability | 0 ^b^ | 0 ^b^ | 0 ^b^ |
|  | 0.41 | Common | Varying period effect, Decreasing variability | 0 ^b^ | 7 ^b^ | 41 ^b^ |
|  | 0.41 | Common | Varying period effect, Stable variability | 0 ^b^ | 43 ^b^ | 23 ^b^ |
|  | 0.41 | Varying | Common period effect, high variability | 0 ^b^ | 0 ^b^ | 0 ^b^ |
|  | 0.41 | Varying | Common period effect, low variability | 0 ^b^ | 0 ^b^ | 0 ^b^ |
|  | 0.41 | Varying | Varying period effect, Decreasing variability | 1 ^b^ | 17 ^b^ | 46 ^b^ |
|  | 0.41 | Varying | Varying period effect, Stable variability | 0 ^b^ | 47 ^b^ | 30 ^b^ |

^a^ In scenarios where this effect varies between clusters this is the geometric mean effect

^b^ This is the percentage of simulations where the 95% confidence interval contained the group 1 true effect

**S9a: Estimation of intercept between-cluster variance**

Hollow point: Mean estimate, dashed line: IQR of estimates.

4. Varying period effect stable variability

3. Varying period effect decreasing variability

2. Common period effect low variability

1. Common period effect high variability

**B. Varying intervention effect**

4. Varying period effect stable variability

3. Varying period effect decreasing variability

2. Common period effect low variability

1. Common period effect high variability

**A. Common intervention effect**

0.5

1.0

1.5

2.0

Model

Standard

Random Period

Random Intervention

**S9b: Table of mean intercept variance (between-cluster variance) estimates from simulations**

| **Group two Intervention effect log(OR)** | **Group one Intervention effect log(OR) ^a^** | **Intervention effect** | **Period effect** | **Estimated variance** | | |
| --- | --- | --- | --- | --- | --- | --- |
|  |  |  |  | **Standard**  **Model** | **Random**  **Period Model** | **Random Intervention Model** |
| Same as  group 1 log(OR)=0.41 | 0.41 | Common | Common period effect, high variability | 1.79 | 1.79 | 1.79 |
|  | 0.41 | Common | Common period effect, low variability | 0.25 | 0.25 | 0.25 |
|  | 0.41 | Common | Varying period effect, Decreasing variability | 0.38 | 1.78 | 1.08 |
|  | 0.41 | Common | Varying period effect, Stable variability | 1.26 | 1.77 | 1.47 |
|  | 0.41 | Varying | Common period effect, high variability | 1.91 | 1.88 | 1.79 |
|  | 0.41 | Varying | Common period effect, low variability | 0.38 | 0.34 | 0.25 |
|  | 0.41 | Varying | Varying period effect, Decreasing variability | 0.51 | 1.88 | 1.06 |
|  | 0.41 | Varying | Varying period effect, Stable variability | 1.39 | 1.88 | 1.50 |
|  | 0 | Common | Common period effect, high variability | 1.78 | 1.78 | 1.79 |
|  | 0 | Common | Common period effect, low variability | 0.25 | 0.25 | 0.25 |
|  | 0 | Common | Varying period effect, Decreasing variability | 0.37 | 1.77 | 1.07 |
|  | 0 | Common | Varying period effect, Stable variability | 1.26 | 1.78 | 1.48 |
|  | 0 | Varying | Common period effect, high variability | 1.91 | 1.89 | 1.78 |
|  | 0 | Varying | Common period effect, low variability | 0.39 | 0.35 | 0.25 |
|  | 0 | Varying | Varying period effect, Decreasing variability | 0.50 | 1.88 | 1.07 |
|  | 0 | Varying | Varying period effect, Stable variability | 1.37 | 1.87 | 1.49 |
| Different to group 1 log(OR)=1.5 | 0.41 | Common | Common period effect, high variability | 2.06 | 2.07 | 2.09 |
|  | 0.41 | Common | Common period effect, low variability | 0.51 | 0.51 | 0.48 |
|  | 0.41 | Common | Varying period effect, Decreasing variability | 0.76 | 1.85 | 1.08 |
|  | 0.41 | Common | Varying period effect, Stable variability | 1.53 | 1.84 | 1.49 |
|  | 0.41 | Varying | Common period effect, high variability | 2.13 | 2.11 | 1.94 |
|  | 0.41 | Varying | Common period effect, low variability | 0.59 | 0.53 | 0.27 |
|  | 0.41 | Varying | Varying period effect, Decreasing variability | 0.84 | 1.94 | 1.06 |
|  | 0.41 | Varying | Varying period effect, Stable variability | 1.60 | 1.95 | 1.49 |

^a^ In scenarios where this effect varies between clusters this is the geometric mean effect

True variation of intercept is 1.79 for scenarios with a common period effect with high variability and both scenarios with varying period effect, and 0.25 for the scenario with common period effect and low variability.

**S10a: Figure of Type 1 error**

4. Varying period effect stable variability

3. Varying period effect decreasing variability

2. Common period effect low variability

1. Common period effect high variability

**B. Varying intervention effect**

4. Varying period effect stable variability

3. Varying period effect decreasing variability

2. Common period effect low variability

1. Common period effect high variability

**A. Common intervention effect**

0.0

0.2

0.4

0.6

0.8

Type 1 error

Model

Standard

Random Period

Random Intervention

**S10b: Table of Type 1 error rate of simulations**

| **Group two Intervention effect log(OR)** | **Group one Intervention effect log(OR) ^a^** | **Intervention effect** | **Period effect** | **Type 1 error** | | |
| --- | --- | --- | --- | --- | --- | --- |
|  |  |  |  | **Standard**  **Model** | **Random**  **Period Model** | **Random Intervention Model** |
| Same as  group 1 log(OR)=0.41 | 0.41 | Common | Common period effect, high variability | 4 | 4 | 3 |
|  | 0.41 | Common | Common period effect, low variability | 5 | 5 | 5 |
|  | 0.41 | Common | Varying period effect, Decreasing variability | 93 | 7 | 26 |
|  | 0.41 | Common | Varying period effect, Stable variability | 91 | 7 | 12 |
|  | 0.41 | Varying | Common period effect, high variability | 78 | 14 | 5 |
|  | 0.41 | Varying | Common period effect, low variability | 75 | 12 | 4 |
|  | 0.41 | Varying | Varying period effect, Decreasing variability | 91 | 6 | 23 |
|  | 0.41 | Varying | Varying period effect, Stable variability | 93 | 5 | 11 |
|  | 0 | Common | Common period effect, high variability | 4 | 3 | 4 |
|  | 0 | Common | Common period effect, low variability | 5 | 5 | 5 |
|  | 0 | Common | Varying period effect, Decreasing variability | 91 | 4 | 20 |
|  | 0 | Common | Varying period effect, Stable variability | 92 | 5 | 12 |
|  | 0 | Varying | Common period effect, high variability | 78 | 15 | 5 |
|  | 0 | Varying | Common period effect, low variability | 74 | 17 | 7 |
|  | 0 | Varying | Varying period effect, Decreasing variability | 90 | 5 | 18 |
|  | 0 | Varying | Varying period effect, Stable variability | 91 | 5 | 12 |
| Different to group 1 log(OR)=1.5 | 0.41 | Common | Common period effect, high variability | 100 ^b^ | 100 ^b^ | 100 ^b^ |
|  | 0.41 | Common | Common period effect, low variability | 100 ^b^ | 100 ^b^ | 100 ^b^ |
|  | 0.41 | Common | Varying period effect, Decreasing variability | 100 ^b^ | 93 ^b^ | 59 ^b^ |
|  | 0.41 | Common | Varying period effect, Stable variability | 100 ^b^ | 57 ^b^ | 77 ^b^ |
|  | 0.41 | Varying | Common period effect, high variability | 100 ^b^ | 100 ^b^ | 100 ^b^ |
|  | 0.41 | Varying | Common period effect, low variability | 100 ^b^ | 100 ^b^ | 100 ^b^ |
|  | 0.41 | Varying | Varying period effect, Decreasing variability | 99 ^b^ | 83 ^b^ | 54 ^b^ |
|  | 0.41 | Varying | Varying period effect, Stable variability | 100 ^b^ | 53 ^b^ | 70 ^b^ |

^a^ In scenarios where this effect varies between clusters this is the geometric mean effect

^b^ This is the percentage of simulations that rejected at the 5% level the null hypothesis that the intervention effect was equal to the true group 1 intervention effect.

**S11: Deworming trial data cleaning**

We performed the same data cleaning steps used for the reanalysis of the trial [1, 2] to the data available at:

<https://dataverse.harvard.edu/dataset.xhtml?persistentId=doi:10.7910/DVN/28038>

(data downloaded 19/11/2015)

These were as follows:

1. Carry forward missing school ID
2. Remove observations after a pupil moved school
3. Remove observations of pupils who have died, finished school, or moved to secondary school
4. Recode pupil drop out as unattended
5. Remove unscheduled visits.
6. Remove pupils from the data if they were never observed in school during the 2 years
7. Remove visits that had more the 70% missing attendance data for pupils.

**References:**

1. Davey, C., et al., *Re-analysis of health and educational impacts of a school-based deworming programme in western Kenya: a statistical replication of a cluster quasi-randomized stepped-wedge trial.* International Journal of Epidemiology, 2015. **44**(5): p. 1581.

2. Aiken, A.M., et al., *Re-analysis of health and educational impacts of a school-based deworming programme in western Kenya: a pure replication.* International Journal of Epidemiology, 2015. **44**(5): p. 1572.
